# Improving Iodine Intake in Rural Haiti through Social Enterprise: A Cross-Sectional Study in the Central Plateau

**DOI:** 10.3390/nu15051092

**Published:** 2023-02-22

**Authors:** Nora Barloggio, Fr. Herald Jean, Ben Ali Thelus, Pierre Jocenais, Gilbert J. Wirth, Neil Boothby, Kate Schuenke-Lucien, Jessica Rigutto-Farebrother

**Affiliations:** 1 Human Nutrition Laboratory, Institute for Food, Nutrition and Health, ETH Zürich, CH-8092 Zürich, Switzerland; 2 Unité Diocésaine d’Enseignement de Recherche et de Service Hinche, Université Notre Dame d’Haiti, Route Nationale 3, Sapaterre, Hinche BP 1594, Haiti; 3 Kwasans Foundation, Chesapeake, VA 23320, USA; 4 Global Center for the Development of the Whole Child, University of Notre Dame, South Bend, IN 46556, USA; 5 Laboratory for Nutrition and Metabolic Epigenetics, Institute for Food, Nutrition and Health, ETH Zürich, CH-8092 Zürich, Switzerland

**Keywords:** iodine deficiency, Haiti, remote communities, urinary iodine concentration, thyroglobulin, school-age children, women of reproductive age, social enterprise scheme, iodized salt, global child health

## Abstract

Iodine intake in Haiti has increased in recent years thanks to the “Bon Sel” social enterprise approach to salt fortification and distribution by the market segment. However, it was uncertain whether this salt reached remote communities. This cross-sectional study aimed to assess the iodine status of school-age children (SAC) and women of reproductive age (WRA) in a remote region of the Central Plateau. A total of 400 children (9–13 years) and 322 women (18–44 years) were recruited through schools and churches, respectively. Urinary iodine (UIC) and urinary creatinine (UCC) concentrations were measured in spot samples, and thyroglobulin (Tg) on dried blood spots. Their iodine intake was estimated, and dietary information collected. The median (IQR) UIC in SAC was 130 µg/L (79–204, *n* = 399), and in WRA, 115 µg/L (73–173, *n* = 322). The median (IQR) Tg in SAC was 19.7 µg/L (14.0–27.6, *n* = 370), and in WRA, 12.2 µg/L (7.9–19.0, *n* = 183); 10% of SAC had Tg > 40 µg/L. Estimated iodine intake was 77 µg/day and 202 µg/day in SAC and WRA, respectively. Iodized table salt was rarely consumed, though bouillon was used daily; this is hypothesized to be a major contributor to dietary iodine intake. Iodine intake in this remote region seems to have improved considerably since the 2018 national survey, though SAC remain at risk. These results point to the potential effectiveness of using social business principles to deliver humanitarian solutions.

## 1. Introduction

Iodine is an essential micronutrient required to produce thyroid hormones which are needed for brain development and growth. Habitual adequate iodine intake is of critical importance in children, and in women of childbearing age who may be preparing for pregnancy. The recommended daily allowance (RDA) in children above 8 years old and adults is 150 µg/L according to the WHO [1]. In population studies of an adequate sample size [2], this is denoted by a median urinary iodine concentration (UIC) of 100–199 µg/L in school-age children (SAC) [3], in which variation due to factors such as hydration is assumed to even out [4]. UIC indicates the most recent iodine intake, whereas the thyroid prohormone thyroglobulin (Tg) indicates iodine intake over a longer period and is therefore complementary to UIC in population studies [1,5,6]. A median Tg concentration <13 µg/L and/or <3% of Tg values >40 µg/L suggests adequate habitual iodine intake in children and adults [1,7,8].

Haiti is a very complex country with environmental, political, and economic challenges. It is particularly vulnerable to natural disasters and climate change, with related risks of water shortage and sanitation risks that exacerbate food insecurity, which is a permanent problem in the country [9]. Historically, iodine intake in Haiti has been low [10,11,12,13]; the usual Haitian diet is generally low in native iodine [9,14], and intake of iodine-rich foods such as seafood, milk, and dairy are limited and not accessible for the whole population. Knowledge about and consumption of iodized salt is low, and locally produced, small-pan salt is non-iodized and frequently dirty [3,15,16]. Data on iodine intake and status in the Haitian population are scarce and, except for the national survey, not representative. The median UIC (mUIC) concentration in the 2018 UNICEF national survey was 77 µg/L (95% CI 74; 79) in women of reproductive age (WRA) and 74 µg/L (95% CI 68; 79) in pregnant women, respectively [16]. The rural regions had a lower mUIC in WRA (62 µg/L; 95% CI 59; 66; *p* < 0.0001), though sample sizes in these regions were insufficient to draw firm conclusions about habitual iodine intake [4,16].

Universal salt iodization (USI) has been adopted by over 120 countries globally [3,15] and is considered as the safest, most cost-effective, and sustainable strategy to control iodine deficiency in nearly all affected regions and population groups [1,17,18,19,20]. However, while legislation was passed in 2017, the iodized salt policy in Haiti has yet to be implemented. To address this, the “Bon Sel Initiative”, owned and operated by the Congregation de St. Croix, Haiti, nestled within the University of Notre Dame (US) Global Center for the Development of the Whole Child (GC-DWC) and in partnership with the Haitian Ministry of Health, inaugurated a commercial salt processing factory in 2014 that remains the only Haitian supplier of iodized salt. Bon Sel is a non-profit entity which employs a social enterprise business model that allows small, local salt producers to make a strong market contribution by selling to a local processor who can, in turn, purify, fortify and package the salt for sale under the Bon Sel Dayiti brand [21]. This approach has led to increased presence of iodized salt in three market segments: processed foods (bouillon, bread), food service (school meal programs) and retail (packaged sachets). Sales are principally in the West Department, since shipments to the food processing and foodservice market segments are highly centralized in Port-au-Prince, and challenges linked to the volatile situation in Haiti hamper effective market pervasion.

The present study therefore aims to assess the iodine status of two priority-for-intervention population subgroups in a remote region of the Central Plateau. Updated statistics for this region are needed to support the Bon Sel Initiative approach and the development and implementation of appropriate iodine interventions in the area. Based on previous data, we hypothesized that the mUIC for both SAC and WRA would indicate insufficient habitual iodine intake.

## 2. Materials and Methods

This report follows the STROBE reporting guidelines for cross-sectional studies with nutrition extension [22].

### 2.1. Study Site and Subjects

**Figure 1:**
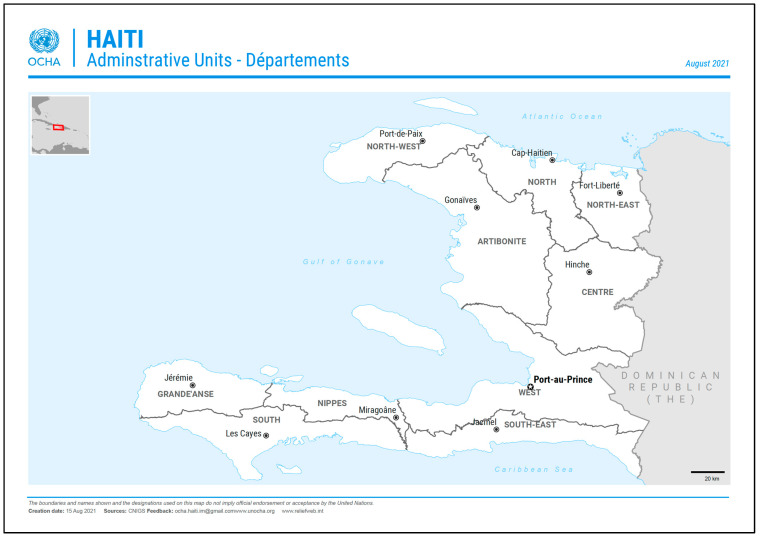
Departments of Haiti, including the capital city, Port-au-Prince, and location of the center department, whose capital is Hinche. Study villages are located in the remote regions around Hinche. (Figure provided by the reliefweb service of OCHA.).

This study was conducted in the region of Hinche, situated in the Central Plateau, Haiti, which has a total population of about 712,000, mainly residing in the principal department city of Hinche (Figure 1). The remote-dwelling population is small, and not precisely known. Outside of the city, the local economy is largely based on farming, the raising of livestock, and small trade. The main foods consumed in Hinche are peas, rice, maize, plantain, poultry, goats, and pork. The use of iodized table salt is rare, and a survey of bouillon cubes available at local markets did not specify iodized salt on their outer packaging. In a 2018 survey, the mUIC for the Central Department (*n* = 169) was reported to be 64 µg/L in WRA [16].

Male and female SAC aged between 9 and 13 years were recruited from local schools within the local diocese GC-DWC network. WRA aged 18 to 44 years were recruited from local churches. One week before the study team was due to visit each study site, information sessions were held by the study coordinator at the participating schools and churches to inform and invite eligible members of the community to participate in the study. Only interested participants returned to the study sites on sample collection day.

Inclusion criteria for all subjects were: (i) residence within the study area for ≥12 months; (ii) general good health as assessed by no reported treatment for chronic disease; (iii) no known history of goiter or other thyroid diseases; (iv) no exposure to iodine-containing contrast agent or medication within the last year. Additional criteria for women stated that WRA should not currently be pregnant or breastfeeding; this was assessed by asking the women. Women did not have to be the parents of the participating child due to the feasibility of recruitment. Participation was voluntary, and written informed consent was obtained from every subject (or parent/caretaker for SAC) at study entry. In addition, each child was asked to provide oral consent to participate in a semi-private setting. All data collected were anonymized before analysis.

Ethical approval was granted by the Comité National de Bioéthique, Haiti, as well as the Ethics Committee of the ETH Zürich, Switzerland. The study was conducted in compliance with a predefined protocol (ClinicalTrials.gov Identifier: NCT05275452), the current version of the Declaration of Helsinki, the ICH-GCP, and the Human Research Act (HRA) as well as other local legal and regulatory requirements. The Human Nutrition Laboratory at ETH Zürich led the study and was responsible for the setup and training, and laboratory and data analysis. The Université Notre Dame d’Haïti UDERS (UND UDERS) of Hinche was the local principal investigator, and responsible for conducting the study in Haiti according to the protocol. The partner institutions of the University of Notre Dame, USA, and the Kwasans Foundation provided administrative support in the field.

### 2.2. Study Design

The study design was cross-sectional. Data were collected between January and June 2022. After a brief interview with the subjects (or their parent(s)/caretaker in the case of SAC), the investigators assessed the validity of the inclusion criteria and subsequent enrollment into the study. Weight was measured to the nearest 0.1 kg, and height to the nearest 0.5 cm using standard anthropometric techniques. A questionnaire was administered to all subjects to gather data on their general dietary habits, including questions about the household total consumption of salt, consumption of iodized salt, consumption of processed foods and foods with high native iodine content including dairy foods, fish, eggs, bread or bouillon, as well as manioc consumption, dietary supplement use and current cigarette smoking in women (Supplemental Tables S1 and S2). All questionnaires and study material were translated from the original (English and French) into the local language (Créole) and delivered by a trained local study team consisting of biomedical sciences graduates from the UND-UDERS Hinche.

### 2.3. Sample Collection

#### 2.3.1. Spot Urine Sample

A spot urine sample (not the first morning void) was collected from all subjects to assess urinary iodine concentration (UIC) and urinary creatinine concentration (UCC). The subjects provided about 50 mL of fresh midstream urine into an iodine-free clean plastic cup. Afterwards, all urine samples were aliquoted (at least 1 mL per aliquot) into three 2.0 mL Eppendorf tubes (Eppendorf, Germany), and frozen at −20 °C until analysis.

#### 2.3.2. Capillary Blood Spot Samples

In addition to urine samples, subjects provided a dried capillary whole-blood spot (DBS) sample for the assessment of thyroglobulin (Tg). DBS samples were collected using a sterile single-use lancet by a student nurse duly trained in collection techniques. For every subject, six blood drops (about 50 µL) were collected by finger prick and directly spotted onto filter paper cards (IDBS-226, Perkin Elmer, CT, USA). These were successively dried at room temperature, placed in sealed plastic bags with a desiccant, and frozen at −20 °C until analysis.

Using the same finger prick and a Hemocue meter (Hemocue AB, Ängelholm, Sweden), hemoglobin (Hb) was measured to control for the iron status of the subjects, a potential confounder to thyroid function indicators and applicable in the case of iodine deficiency.

### 2.4. Laboratory Analysis

Sample collection materials were tested for iodine contamination before the start of the study and certified as iodine-free. Urine and DBS collected in situ in Haiti were sent in two cold shipments to ETH Zurich, where all laboratory procedures were carried out, since no appropriate laboratory set-up at The University of Notre Dame, Haiti, currently exists. However, the cold chain in the second shipment could not be guaranteed, and some samples were lost.

#### 2.4.1. Urinary Iodine Concentration

Iodine concentration in urine was analyzed using the kinetic spectrophotometric Sandell–Kolthoff method modified from Pino et al. [23,24]. The inter-assay variation determined at the Laboratory of Human Nutrition, ETH Zurich, was 8.2% at 60 ± 4.9 μg/L for lower urine control samples and 5.2% at 180 ± 9.3 μg/L for higher urine control samples. Adequate iodine status in SAC and WRA was defined as a median UIC >100 µg/L [1].

#### 2.4.2. Urinary Creatinine Concentration

UCC was analyzed using the modified Jaffé method [25,26]. As an external control, the human urine based Liquichek Chemistry control, Level 1 (4.04 mmol/L–6.10 mmol/L) and Level 2 (10.8 mmol/L–13.6 mmol/L) (Biorad, Hercules, CA, USA) were used. The inter-assay variation was 4.3% at 100 ± 4.3 µg/L for the first level and 2.9% at 205 ± 6 µg/L for the second urine control sample level.

#### 2.4.3. Dried Blood Spot Analysis

DBS-Tg was analyzed with an enzyme-linked immunosorbent assay (ELISA). Serum control samples (Liquicheck Tumor Marker Control, LOT.19950 and LOT.19970; Bio-Rad, Hercules, CA, USA) were used as standards for the assay. In-house DBS-samples were prepared and used as the quality control. The inter-assay variation was 22% at 11.1 ± 2.5 μg/L for the lower quality control and 12% at 71.9 ± 8.9 μg/L for the higher quality control (in line with other studies using this method, e.g., [17]). The reference interval for school-age children is established at 4–40 µg/L Tg [1], with a median Tg concentration <13 µg/L and/or <3% of Tg values >40 µg/L used to indicate population adequate iodine status in children [7]. No reference range has currently been defined for non-pregnant, non-lactating adult women.

### 2.5. Statistical Analysis

The sample size required to estimate the expected prevalence of inadequate iodine intake was calculated according to Lwanga et al. [27]. The estimation of the proportion of individuals with intake below the EAR (estimated average requirement) of 50%, with a power (β) of 80%, relative precision (α) of 5% and a 95% confidence interval (CI), determined a sample size of 384 subjects. To account for dropouts, a final sample size of 400 SAC and 400 WRA was defined; this sample size is generally considered to be sufficient in cross-sectional iodine studies to establish a population’s iodine status [2].

Data processing and statistical analysis were performed using Microsoft Excel, version 16.59, and IBM SPSS Statistics (IBM Company, Armonk, NY, USA), version 27. All data were anonymized and checked thoroughly for data entry errors. The data were graphically evaluated for normal distribution through histograms, Q-Q plots, and P-P plots and by using the Kolmogorov–Smirnov test and the Shapiro–Wilk test. Unless specified, all normally distributed data are described as mean ± SD, and nonparametric data are described as median (IQR).

Weight and height were used to calculate body mass index (BMI). According to the age- and sex-specific 85th and 95th BMI percentiles of the WHO, childhood overweight and obesity were defined [28]. For women of reproductive age, overweight was defined as BMI ≥ 25 kg/m^2^, and obesity as BMI ≥ 30 kg/m^2^ [29].

Daily iodine intake was estimated from spot UIC and creatinine concentration and corrected using the urinary creatinine excretion (UCE) reference value based on age, sex and weight. For school-age children, the reference values of Remer et al. were used [30]. For non-Hispanic black women, the references suggested by Barr et al. were applied [31]. Creatinine reference values for different age ranges were available as mg/dL. They were transformed into g·L^−1^·d^−1^ by assuming a standard weight of 60 kg (for an average woman of 160 cm with a BMI of 22) and a urine volume of 1.5 L [32,33]. Estimated urinary iodine excretion (eUIE) was used to calculate iodine intake, assuming an estimated iodine bioavailability of 92% [34]:

$$eUIE\;\left(\;µg\cdot day^{-1}\right)=\frac{UIC\;\left(µg\cdot L^{-1}\right)}{UCC\;\left(g\cdot L^{-1}\right)}\;\cdot UCE\;reference\;value\;\left(g\cdot day^{-1}\right)$$

$$Iodine\;intake\;\left(\;µg\cdot day^{-1}\right)=UIE\;\left(\;µg\cdot day^{-1}\right)\cdot bioavailability$$

Regression analysis, using dietary consumption data as relevant covariates, was used to assess the association between UIC and other secondary outcome parameters. Spearman’s rho was used to test nonparametric associations with ordinal data. Data were bootstrapped (*n* = 1000) where applicable to obtain robust confidence intervals. Any applicable group differences for continuous variables were tested by using Wilcoxon or Mann–Whitney U tests, or one-way ANOVA with Bonferroni post hoc correction. General univariate linear models were applied to test for dietary predictors of UIC and DBS-Tg. Data were transformed to the natural logarithm before analysis. No data were imputed for missing data. The significance level for all tests was set at *p* < 0.05.

## 3. Results

A total of 400 school-age children were recruited from eight out of the 11 villages included in the study, whereas 322 women were sampled from ten different villages (Supplemental Table S3). Following the information sessions one week before enrollment, only persons interested in participating presented for enrollment. All subjects who presented were eligible. Two WRA withdrew their consent due to refusal to conduct the finger prick sample. General characteristics of all subjects at inclusion are shown in Table 1. The mean hemoglobin concentrations did not indicate risk of anemia. However, 50.7% SAC and 37.1% of WRA had an Hb below the 11.5 g/dl and 12 g/dl thresholds [35], respectively.

### 3.1. Iodine Status

Iodine status is described in Table 2.

In SAC, an association was found between UIC and age (ρ = −0.130; 95% CI = −0.228, −0.036; *p* = 0.010), iodine intake and BMI (ρ = 0.224; 95% CI = 0.122, 0.313; *p* < 0.001). DBS-Tg was associated with age (ρ =−0.163; 95% CI = −0.257, −0.056; *p* = 0.002), weight (ρ =−0.177; 95% CI = −0.271, −0.071; *p* < 0.001), height (ρ = −0.121, 95% CI = −0.230, −0.019; *p* = 0.020) and consequently, BMI (ρ =−0.179; 95% CI = −0.274, −0.082; *p* < 0.001). In WRA, weight (ρ = 0.153; 95% CI = 0.013, 0.293; *p* = 0.039) and number of children (ρ = 0.306; 95% CI = 0.170, 0.442; *p* < 0.001) were positively associated with DBS-Tg, whereas degree of education (ρ = −0.247; 95% CI = −0.375, −0.102; *p* < 0.001) was inversely associated. No other associations were found.

### 3.2. Dietary Data

Dietary data from questionnaires indicated a regular intake of native iodine-rich foods such as dairy produce, eggs, and fish, as well as regular consumption of foods that have the potential to be fortified with iodine via iodized salt, namely bouillon cubes and bread. Over 90% of SAC and WRA reported at least weekly consumption of these foods (Supplemental Table S4). Manioc root was also consumed once per week or more by 90% of SAC and 93% of WRA (Supplemental Table S4). When asked how many bouillon cubes they consume per meal, 18% (*n* = 57) of women reported an average consumption of three cubes, 46% (*n* = 149) indicated two cubes, and 22% (*n* = 70) one cube. The remaining proportion reported consuming either more than three cubes per meal, reported not using bouillon, or the information was not available. Use of iodized table salt was low; 16.5% (*n* = 53) of women reported household use; 13% (*n* = 43) during cooking and 12% (*n* = 40) at the table. Most (62%; *n* = 201) bought salt for household use at the local market (“Sel du marché”), and 9% (*n* = 30) of women reported using the “Bon Sel Dayiti” iodized salt.

In SAC, an association was found between iodine intake and manioc consumption (ρ = 0.114; 95% CI = 0.019; 0.197; *p* = 0.029). No other associations between iodine intake and SAC dietary data were found. In a univariate general linear model to test for dietary predictors of UIC, fish and manioc root consumption were found to be significant predictors of UIC (β = 0.350; 95% CI −0.020, 0.0796; *p* = 0.049 and β = 0.479; 95% CI 0.030, 0.912; *p* = 0.016). The corrected model with only these two covariates was significant (*p* = 0.031). No significant predictors of Tg were found.

In WRA, associations were found between UIC and bouillon consumption (ρ = 0.154; 95% CI = 0.034, 0.262; *p* = 0.006) and UIC and fish consumption (ρ = 0.138; 95% CI = 0.043, 0.227; *p* = 0.013), and DBS-Tg was lower in women regularly consuming fish (ρ = −0.147; 95%CI = −0.266, −0.028; *p* = 0.049). Women consuming bouillon and fish once or more per week had a greater iodine intake than women consuming these foods less regularly or not at all (ρ = 0.140; 95% CI = 0.021, 0.254; *p* = 0.012 and ρ = 0.162; 95% CI = 0.028, 0.273; *p* = 0.004; respectively). UIC in WRA increased with the average number of bouillon cubes consumed per meal (ρ = 0.117; 95% CI = −0.004, 0.232; *p* = 0.043). No other associations were found, and there were no predictors of UIC and Tg in linear models for WRA.

## 4. Discussion

In this study, we assessed the iodine status of two priority population subgroups of the remote-dwelling population of the Central Plateau. Based on the mUIC of 130 µg/L in SAC, and in WRA, of 115 µg/L, the results did not confirm our initial hypothesis of insufficient iodine intake in either sample, based on the threshold for population habitual adequacy denoted by WHO of 100 µg/L in school-age children [1]. However, 10% of DBS-Tg samples from SAC were >40 µg/L, indicating that despite the UIC there was a risk of inadequate iodine intake. In WRA, this proportion was just over 2%, suggesting adequacy.

For WRA, these results suggest an important improvement in the iodine status in this region compared to the 2018 UNICEF survey [16,37]. This was underlined by correcting the UIC for creatine and thereby estimating daily iodine intake, which rose to an average of just over 200 µg/day, which is above the WHO recommendation but not so high as to risk excessive intake [19]. In SAC, a robust comparison is not available, since previous studies in SAC are generally weak and/or conducted in different regions [10,12,13]. Though an improvement in this population group may also be inferred based on the hydration status of these children and our subsequent iodine intake estimations, adequate habitual iodine intake may be at risk in our sample: the estimated mean daily iodine intake was approximately half that recommended amount. The percentage of children with a DBS-Tg > 40 µg/L also supports this conclusion [7].

Since Haiti has not yet implemented its fortification law requiring the iodization of locally produced salt, to maintain the target of adequate iodine nutrition in this population, it is important to understand the rationale behind it. Based on the ensemble of the available data, and though we cannot prove it through iodine content analysis of locally available products, we believe that bouillon cubes are likely the main vehicle for iodized salt in this population and thus the main contributor to total iodine intake. In contrast to iodized salt, the utilization of bouillon in Haiti is widespread, as was reported in the 2018 national survey and confirmed by dietary questionnaire used for this study. The use of bouillon cubes for mitigating iodine deficiency in Haiti has been promoted by UNICEF [38] and supported by von Oettingen et al. [39]; however, to date, it is unclear how pervasive iodized salt is within all available bouillon cube brands which can vary widely in iodine content [38,39,40]. To support this hypothesis, our study found a greater mUIC in women regularly consuming bouillon compared to those not consuming it; this was reinforced by the positive association found between the average number of bouillon cubes consumed per meal and the UIC. For the SAC, these associations were not found, but instead, children regularly consuming manioc root had a greater mUIC compared to those without a regular consumption. This association may reflect the usual consumption of manioc root with sauces, mashed peas or in soup, which are likely prepared using bouillon cubes, thus resulting in the positive association. However, it is well known that manioc contains cyanogenic glucosides, which are transformed into goitrogenic thiocyanates that can interfere with the normal iodine metabolism by binding to the NIS and blocking iodine uptake from the circulation into the thyroid follicular cells [41], thus increasing its excretion in urine. This may further explain the 10% prevalence of elevated DBS-Tg in this group, which is suggestive of inadequate iodine intake.

All the Bon Sel Initiative salt sold for human consumption contains iodine in the form of KIO_3_ at a concentration of 40 mg/kg. Bon Sel works closely with stakeholders in Haiti, including the government, salt producers, the food industry and national and international NGOs, and supplies salt to several sectors, particularly bakeries, school lunch programs, and bouillon manufacturers. The growth of sales in those sectors, especially in the bouillon manufacturer market, could also support the hypothesis that bouillon is the principal vehicle for iodine in this population. Indeed, the leading brands, representing over 70% of the national market, are, as of late, produced with iodized salt supplied by the Bon Sel Initiative. Since the inauguration of the Bon Sel factory in 2014, at that time producing coarse iodized salt only, manufacturing has expanded to include production of both coarse and fine iodized salt and unfortified salt for industrial use. Annual sales of all types of Bon Sel salt have increased from 100 tons/year to a peak of 5000 tons/year in 2021. Current production of fortified salt is split almost equally between coarse and fine salt, with half of the latter going to Haitian bouillon producers who have been served by Bon Sel since October 2017 (personal communication, James Reimer). This business model provides entry of iodized salt into the foodservice and food-processing segments, thus maintaining coverage if/when retail distribution is prohibitively slow or costly, and even in difficult periods. Despite social unrest, environmental challenges and economic and political upheavals, Bon Sel, as a social enterprise initiative managed and operated by Haitians, has continued distribution of fortified salt throughout.

Though we cannot confirm causation from our study, our data suggest that, if successful, this business model may have implications broader than Haiti alone; many countries could benefit from improving the access of small-scale salt producers to local salt processors and the packaged salt supply chain, whilst ensuring their contribution to national salt iodization policy as well as their livelihoods. This model calls attention to the cost effectiveness and efficiency of leveraging the supply chain and using business principles to improve the prospects of delivering humanitarian solutions.

The dietary questionnaires suggest that most women and children regularly consume dairy products. In general, many animal food products are imported from the neighboring Dominican Republic (personal communication, James Reimer), where a Universal Salt Iodization (USI) program has been in place since 1994, with iodization set between 60 and 100 mg iodine/kg, averaging 75 mg/kg [38]. USI stipulates that all food for human and animal consumption be fortified with iodine via iodized salt [20]. We were not able to obtain any data about the specific importation of goods from the Dominican Republic; however, if our information is correct, dairy products imported from the Dominican Republic may be rich in iodine and could represent an important contribution to the total iodine intake in the Haitian population in this region. More data are needed to confirm this theory.

Iodine interacts with other micronutrients, and in particular, an appropriate iron status is important to ensure the enzymatic activity of thyroid peroxidase, which catalyzes the iodination of tyrosyl residues on Tg in the thyroid colloid [42]. Controlling for iron status in iodine studies where deficiency is suspected is therefore advisable. We found that 37% of WRA and approximately 50% of SAC (taking the two different cut-offs for the different age ranges) may have low Hb concentrations. As shown in previous studies, iron supplementation may be beneficial also in preventing iodine deficiency [43,44,45,46]. In the Haitian context, a follow-up for iron status assessment, involving recommended iron status biomarkers such as ferritin [47], should be considered, especially given the estimated lower iodine intake in children.

This study has several strengths. The combination of data on both SAC and WRA across several villages provide a greater insight into the iodine status in this remote region compared to previous studies, and improves the generalizability of these data to other, similarly remote regions. We assessed iodine status not only via UIC, but also via DBS-Tg, and adjusted the UIC for hydration using urinary creatinine, strengthening the validity of our results, rather than relying on UIC alone.

The limitations of this study include potential selection bias in the recruitment of the subjects and the choice of the study sites. SAC were recruited from schools, which is standard practice in such studies, whereas WRA were recruited via local Catholic churches, taking advantage of existing diocesan links with the study team and the University of Haiti’s Hinche campus. It is likely that some very rural villages are still missing and should also be sampled. We might expect this selection bias to positively influence the iodine status observed in the sample population of this study; however, due to the surprising finding of iodine sufficiency, this is difficult to predict with any certainty. Due to slow recruitment (because of fuel shortages at the time of the study because of ongoing conflicts, and because many eligible women were not present in the villages and rather in the cities to find work), we did not reach the desired sample size for WRA, thereby weakening our conclusions. Many WRA DBS samples were also lost during shipment from Haiti to ETH Zürich for analysis. Though our questionnaire was adequate to assess background dietary intake of native iodine-rich and iodine-fortified foods, the unexpected finding of mUIC >100 μg/L in both groups shifted the focus of the discussion. The goal became to identify the reasons for this improvement. However, the questionnaires lacked sufficient granularity for this purpose. Since almost all children and women reported regular consumption of all food groups included in the questionnaire, it was difficult to make inferences. Though bootstrapping was applied to improve robustness, our data are challenged by unbalanced groups. A more exhaustive and quantitative food frequency questionnaire (FFQ) or a 24 h dietary recall would have provided more comprehensive insight, though they are more difficult to put into practice. Following our market survey prior to the study, we believed that bouillon available locally did not contain iodized salt, and it is thus a limitation that we were not permitted to ship bouillon cubes to ETH Zürich for iodine analysis. Finally, we did not collect household salt to verify iodine content.

## 5. Conclusions

To conclude, our study shows encouraging and promising results. Habitual iodine intake in non-pregnant, non-lactating women living in the Central Plateau, Haiti, seems adequate, and our data suggest a considerable improvement compared to previous studies. While the UIC in children of school age in this region also suggests adequacy, when iodine intake is estimated considering hydration status using creatinine, it is below that recommended by the WHO. Given the importance of assuring iodine intake during critical development periods such as childhood, as well as in pregnancy, lactation and adolescence, further work is required to elaborate on better ways to bring iodine into this population. Since the food processing industry (bakeries and bouillon manufacturing) in Haiti is more consolidated compared to local salt production, it is likely the most effective vehicle for distribution of iodine in this population. The disparity between UIC and estimated iodine intake considering hydration status, as well as intake between women and children, however, requires careful monitoring, which should be implemented without delay.

## Figures and Tables

**Table 1:** General characteristics of the study population.

	School-Aged Children	Women of Reproductive Age
*n*	400	322 ^a^
Age (years) ^b^	11.1 ± 1.2	24 ± 7.6
Male ^c^	152 (40.8)	-
Height (cm)	139.4 ± 8.9	159.8 ± 6.0
Weight (kg)	31.6 ± 6.7	57.3 ± 11.7
BMI (kg/m^2^) ^d^	-	22.4 ± 4.3
Hb (g/dl)	12.3 ± 4.7	12.4 ± 2.7
Household ^e^		
People living in household	-	6 ± 2 (1–14)
Parity	-	1 ± 2 (0–8)
Children living in household	-	1 ± 1 (0–8)
Education ^b^		
None	-	14 (4.3%)
Primary	-	79 (24.5%)
Secondary	-	213 (66.1%)
Advanced (university)	-	14 (4.3%)
Smoking	-	6 (1.9%)

^a^ The desired sample size for WRA was not achieved due to slow recruitment. ^b^ Mean ± SD. ^c^ *n* (%). ^d^ BMI-for-age is applied in children. These values are presented in the Supplementary Materials. All values fell between the 15th and the 50th percentiles, which indicates the normal range defined by the WHO [36]; however, the values were closer to the 15th percentile, indicating a propensity to thinness. ^e^ *n* = 321. Data expressed as mean ± SD rounded to a whole person and (range). BMI, body mass index; Hb, hemoglobin; SAC, school-age children; WRA, women of reproductive age.

**Table 2:** Iodine status by population group.

	*n*	SAC	*n*	WRA
UIC (µg/L)	399	130 (79–204)	322	115 (73–173)
UCC (mg/dL)	399	89 (57–130)	322	131 (88–182)
UIC/creatinine ratio (µg/g)	399	141 (101–219)	322	88 (63–133)
Estimated iodine intake (µg/day) ^b^	373	77 (51–116)	320	202 (144–301) ^a^
DBS-Tg (µg/L)	370	19.7 (14.0–27.6)	183 ^c^	12.2 (7.9–19.0)
% > 40 µg/L	38	10.0%	5	2.2%

Data are expressed as median (IQR). A bootstrapped *n* = 1000 was applied. ^a^ The creatinine references for different age ranges were available as mg/dL. They were transformed into g·L^−1^·d^−1^ by assuming a standard weight of 60 kg (for an average woman of 160 cm with a BMI of 22) and a urine volume of 1.5 L [32,33]. ^b^ *n* differs due to missing data points. ^c^ Some 130 DBS-cards for the thyroglobulin analysis deteriorated during transit. DBS, dried blood spot; Tg, thyroglobulin; UCC, urinary creatinine concentration; UIC, urinary iodine concentration.

## Data Availability

Data will be made available at reasonable request.

## Supplementary Materials

The following supporting information can be downloaded at: https://www.mdpi.com/article/10.3390/nu15051092/s1: Table S1. Dietary questionnaire administered to the school-age child and/or their parent or caretaker; Table S2. Dietary questionnaire administered to the women of reproductive age; Table S3. Distribution of the study populations across the 11 villages of the Central plateau included in the study; Table S4. Food consumption data from dietary questionnaires.
