# Supplementary material for: Improving Iodine Intake in Rural Haiti through Social Enterprise: A Cross-Sectional Study in the Central Plateau
Source: Nutrients. 2023 Feb 22;15(5):1092. doi: 10.3390/nu15051092 (PMC10005509; doi:10.3390/nu15051092)
Supplement: Supplementary file 1 [file nutrients-15-01092-s001.zip › nutrients-2116173-supplementary.pdf]

nutrients

Supplementary Materials

Improving Iodine Intake in Rural Haiti through Social Enterprise: A Cross-Sectional Study in the Central Plateau

Nora Barloggio [1,†], Fr. Herald Jean [2,†], Ben Ali Thelus [2], Pierre Jocenais [2], Gilbert J. Wirth Jr. [3], Neil Boothby [4], Kate Schuenke-Lucien [4] and Jessica Rigutto-Farebrother [1,4,5]*

Table S1. Dietary questionnaire administered to the school-age child and/or their parent or caretaker.

ETH zürich — UNIVERSITY OF NOTRE DAME — UNIVERSITE NOTRE DAME D'HAITI

QUESTIONNAIRE-CRF SCHOOL-AGE CHLDREN: ARCH

Assessing the prevalence of iodine deficiency in Remote communities of the Central plateau, Haiti: a cross-sectional study

PAGE 1

Subject code (ID label): I__I__I / I__I__I / I__I__I   Date of interview (DD/MM/YY): I__I__I / I__I__I / I__I__I

Interviewer name	Inclusion criteria fulfilled? (See * marked questions)	Consent signed?
	YES ☐ NO ☐ DK ☐	YES ☐ NO ☐ DK ☐

If yes, please continue.   **In form below, DK= Don't know**

Interview

Date of birth (DD/MM/YY): I__I__I / I__I__I / I__I__I   Age (years): I__I__I   DK ☐

Gender: Male ☐ Female ☐   School years completed: I__I__I

Does the child take vitamin supplements   YES ☐ NO ☐ DK ☐

Does the child regularly (once a week or more) consume any of the following foods?

Milk, Yoghurt, Cheese or other dairy foods YES ☐ NO ☐ DK ☐   Bouillon? YES ☐ NO ☐ DK ☐

Fresh, tinned or dried fish   YES ☐ NO ☐ DK ☐   Eggs   YES ☐ NO ☐ DK ☐

Bread bought from a local bakery YES ☐ NO ☐ DK ☐   Manioc root   YES ☐ NO ☐ DK ☐

Interviewer's comments or any additional information:

____

____

____

____

Table S2. Dietary questionnaire administered to the women of reproductive age.

ETH zürich UNIVERSITY OF NOTRE DAME UNIVERSITE NOTRE DAME D'HAITI

QUESTIONNAIRE-CRF WOMEN: ARCH

Assessing the prevalence of iodine deficiency in Remote communities of the Central plateau, Haiti: a cross-sectional study

PAGE 1

Subject code (ID label): I__I__I / I__I__I / I__I__I   Date of interview (DD/MM/YY): I__I__I / I__I__I / I__I__I

Interviewer name	Inclusion criteria fulfilled?<br>(See * marked questions)	Consent signed?
	YES ☐ NO ☐ DK ☐	YES ☐ NO ☐ DK ☐

If yes, please continue.   **In form below, DK= Don't know**

Interview

Date of birth (DD/MM/YY): I__I__I / I__I__I / I__I__I   Age (years): I__I__I   DK ☐

How many people live in her household?   Adults I__I__I   Children I__I__I__I

How many children has the woman given birth to?   I__I__I

How many children currently live with the mother at home?   I__I__I

Highest degree of education:

☐ No schooling completed   ☐ Primary school   ☐Secondary school   ☐College/University

Does the woman: smoke? YES ☐ NO ☐ DK ☐   Take vitamin supplements? YES ☐ NO ☐ DK ☐

Does the woman regularly (once a week or more) consume any of the following foods?

Milk, Yoghurt, Cheese or other dairy foods: YES ☐ NO ☐ DK ☐   Bouillon: YES ☐ NO ☐ DK ☐

If yes, how many bouillon cubes/sachets in an average meal (e.g. a stew):________________________________

Fresh, tinned or dried fish   YES ☐ NO ☐ DK ☐   Eggs   YES ☐ NO ☐ DK ☐

Bread bought from a local bakery   YES ☐ NO ☐ DK ☐   Manioc root   YES ☐ NO ☐ DK ☐

Is the salt currently used at home iodized salt?   YES ☐ NO ☐ DK ☐

If YES, is the salt: a) used for cooking: YES ☐NO ☐   b) used at the table: YES ☐ NO ☐   c) DK ☐

Brand name of salt currently used at home: ……………………………………………………………………………DK ☐

Interviewer's comments or any additional information:

____

____

____

____

Table S3. Distribution of the study populations across the 11 villages of the Central plateau included in the study.

Village		SAC (n = 400)		WRA (n = 322)	
		n	%	n	%
1.	Layaye	46	11.5	30	9.3
2.	Labegue	31	7.8	6	1.9
3.	Sapaterre	110	27.5	-	-
4.	Thomonde	43	10.8	20	6.2
5.	Colladère	89	22.3	6	1.9
6.	Carissade	69	17.3	31	9.6
7.	Cerca-La-Source	-	-	43	13.4
8.	Cerca-Carvajal	12	3.0	16	5.0
9.	Lospalis	-	-	93	28.9
10.	Maissade	-	-	36	11.2
11.	Thomassique	-	-	41	12.7

Note: the distribution is not equal across the villages and caution is needed in the interpretation of the findings related to differences across the centers where the two study populations come from.

Table S4. Food consumption data from dietary questionnaires.

Foods consumed ≥ 1/week	SAC		WRA	
	%	n[1]	%	n[1]
Milk, yoghurt, cheese or other dairy foods	92.5	370	95.7	308
Bouillon	96.3	385	93.5	301
Fresh, tinned, or dried fish	91.8	367	96.0	309
Eggs	94.3	377	98.4	317
Bread bought from a local bakery	97.3	389	98.1	316
Manioc root	90.3	361	94.1	303
Takes vitamin supplements	2.0	8	4.3	14

[1] n does not correspond to total number of subjects recruited due to incomplete reporting in questionnaires. No data were imputed.
